# *Lactobacillus rhamnosus* GG ameliorates DON-induced intestinal damage depending on the enrichment of beneficial bacteria in weaned piglets

**DOI:** 10.1186/s40104-022-00737-9

**Published:** 2022-08-12

**Authors:** Yongsong Bai, Kaidi Ma, Jibo Li, Zhongshuai Ren, Jing Zhang, Anshan Shan

**Affiliations:** 1 Institute of Animal Nutrition, Northeast Agricultural University, Harbin, 150030 P. R. China; 2 College of Animal Sciences, Jilin University, Key Laboratory of Zoonosis Research, Ministry of Education, Changchun, 130062 P. R. China

**Keywords:** Deoxynivalenol, Gut microbiota, Intestinal inflammation, *Lactobacillus rhamnosus* GG, Piglets

## Abstract

**Background:**

Deoxynivalenol (DON) is one of the most common environmental pollutants that induces intestinal inflammation and microbiota dysbiosis. *Lactobacillus rhamnosus* GG (LGG) is a probiotic that not only has anti-inflammatory effects, but also shows protective effect on the intestinal barrier. However, it is still unknown whether LGG exerts beneficial effects against DON-induced intestinal damage in piglets. In this work, a total of 36 weaned piglets were randomized to one of four treatment groups for 21 d. The treatment groups were CON (basal diet); LGG (basal diet supplemented with 1.77 × 10^11^ CFU/kg LGG); DON (DON-contaminated diet) and LGG + DON (DON-contaminated diet supplemented with 1.77 × 10^11^ CFU/kg LGG).

**Result:**

Supplementation of LGG can enhance growth performance of piglets exposed to DON by improving intestinal barrier function. LGG has a mitigating effect on intestinal inflammation induced by DON exposure, largely through repression of the TLR4/NF-κB signaling pathway. Furthermore, supplementation of LGG increased the relative abundances of beneficial bacteria (e.g., *Collinsella*, *Lactobacillus*, *Ruminococcus_torques_group* and *Anaerofustis*), and decreased the relative abundances of harmful bacteria (e.g., *Parabacteroides* and *Ruminiclostridium_6*), and also promoted the production of SCFAs.

**Conclusions:**

LGG ameliorates DON-induced intestinal damage, which may provide theoretical support for the application of LGG to alleviate the adverse effects induced by DON exposure.

## Introduction

Mycotoxins are prevalent and inevitable environmental pollutant. They are secondary metabolites produced by fungi species and are widespread in cereal grains and predominantly found in wheat, maize, barley, oat, rice and processing by-products of these crops [1]. Cereals are a major carbohydrate source that provide energy and nutrition for humans and animals. In the case of the agricultural practices, about 60% of cereals are contaminated with mycotoxins and this seems to be unavoidable [2]. Therefore, mycotoxins pose serious threats to human and animal health due to its intestinal toxicity, immunotoxicity and neurotoxicity [3, 4].

Deoxynivalenol (DON) is one of the most common and dangerous mycotoxins. The gastrointestinal tract represents the primary target organ after ingestion DON-contaminated feed. In many animal species, acute exposure to DON can cause vomiting, while prolonged exposure to low doses of DON can cause anorexia and weight loss [5]. DON exposure also causes intestinal inflammation, impairs intestinal barrier integrity and reshapes gut microbial structure [6, 7]. Among domestic animals, different species of animals have different sensitivity to DON, and pigs are the most sensitive species [8]. A number of studies have demonstrated that piglets fed DON-contaminated diets can reduce the growth performance causing severe economic losses in the livestock industry [9–11]. Therefore, an urgent demand exists for researchers to find a substance that has a potential to be used as an animal feed ingredient to counteract harmful effects of DON in animals and avoid or reduce economic losses.

*Lactobacillus rhamnosus* GG (LGG) is one of the most-widely used probiotics. Several studies have revealed LGG has anti-inflammatory effects [12], and can be used to prevent and/or treat several diseases, including diarrhea and atopic dermatitis [13, 14]. In addition, LGG can not only prevent intestinal epithelial damage and apoptosis, but also maintain the barrier function [15–18]. It is noteworthy that LGG is able to attach to intestinal mucosa and transiently colonize the intestinal tract [19], which indicates the potential therapeutic application of LGG in intestinal health.

The objective of this experiment was to explore the effect of LGG on the intestinal health of weanling piglets exposed to DON, providing the foundation for further research on the toxicity mechanism of DON and for developing an effective treatment strategies for DON.

## Materials and methods

### Preparation of freeze-dried bacteria and toxins

LGG (ATCC53103) was provided by the China Center of Industrial Culture Collection (CICC, Beijing, China). The bacteria were grown anaerobically in MRS medium (Aoboxing, Beijing, China). After 12 h, the bacteria cells were obtained through centrifugations and washed. The detailed information was described in our previous research [20, 21]. The bacteria were lyophilized for 48 h using lyophilizer (Telstar, Terrassa, Spain). Trehalose (44.4%) was used as the cryoprotectant [22]. The viability of the freeze-dried bacteria was 1.77 × 10^11^ CFU/g and was stored at -80 °C until further use. Bacterial viability and contamination were confirmed once a week.

*Fusarium graminearum* ACCC 37687 was obtained from the Agricultural Culture Collection of China (ACCC, Beijing, China). The fungal was cultivated in Potato-dextrose Agar (Aoboxing, Beijing, China) medium at 25 °C for 7 d. Then, the strain was inoculated in Potato-dextrose broth (Aoboxing, Beijing, China) medium for 7 d to obtain mature spores. Five hundred grams of corn and 100 mL distilled water were added to 2-L Erlenmeyer flasks, and then sterilized by autoclaving at 121 °C for 20 min. To each flask was added 50 mL spore suspension, and cultured in darkness at 28 °C. After 28 d, the mold contaminated corn was dried, mixed, and crushed for reserve use. For the detection of the concentration of DON, the mold contaminated corn was taken and detected with a commercial ELISA kit (Pribolab, Qingdao, China). The content of DON in the mold contaminated corn was 14.92 mg/kg. Before the treatments, corn in the basal diet was partially replaced with mold contaminated corn to provide 3.11 mg DON/kg diet for the DON and LGG + DON treatments.

### Animals and experimental design

All procedures mentioned in the present study were approved by the Institutional Animal Care and Use Committee of Northeast Agricultural University (NEAU-[2011]-9). Thirty-six 21-day-old barrows (Duroc × Landrace × Large White) were randomized to one of four treatment groups (*n* = 9/group): CON (a basal diet); LGG (a basal diet supplemented with 1.77 × 10^11^ CFU/kg LGG); DON (DON-contaminated diet); LGG + DON (DON-contaminated diet supplemented with 1.77 × 10^11^ CFU/kg LGG). The basal diet met or exceeded the requirements for piglets [23]. The ingredient and nutritional level of the basal diet were listed in Table 1. The concentration of DON in basal and DON-contaminated diets were 208 μg/kg and 3.11 mg/kg, respectively, while that of zearalenone were 35.65 μg/kg and 321.67 μg/kg, respectively. AFB_1_ was not detected in all diets.

**Table 1:** Ingredient and nutritional level of diet (as-fed basis)

Items	Content, %	Items	Content, %
Ingredient		Nutritional level^b^	
Corn	63.30	Net energy, kcal/kg	2565
Full-fat expanded soybean	9.00	Crude protein (CP)	18.24
Peeled soybean meal	13.00	Lysine	1.44
Whey powder	5.00	Methionine	0.42
Fish meal	4.00	Threonine	0.89
Soybean oil	2.00	Calcium	0.74
Lysine (98%)	0.60	Total phosphorus	0.60
Methionine (98%)	0.10	Available phosphorus	0.33
Threonine (98%)	0.20		
Calcium hydrogen phosphate	0.70		
Limestone	0.70		
Salt	0.40		
Premix^a^ (1%)	1.00		

*Note*: ^a^Premix provided the following per kilogram of diet: Cu, 20.2 mg; Zn, 106.5 mg; Se, 0.3 mg; Mn, 3 mg; Fe, 120 mg; I, 0.2 mg; vitamin A, 5000 IU; vitamin D_3_, 1250 IU; vitamin E, 47.5 IU; vitamin K, 2.2 mg; vitamin B_1_, 3.6 mg; vitamin B_2_, 8.0 mg; vitamin B_6_, 4.1 mg; vitamin B_12_, 0.04 mg; pantothenic acid, 18 mg; niacin, 29.7 mg; folate, 1.9 mg and biotin, 0.4 mg.

^b^Nutrient levels were calculated values

Piglets were raised individually in metabolic cages, and the piglets were provided with free access to water and different diets during the 21-day experiment period. Food intake and body weight were recorded to calculate average daily food intake (ADFI) and average daily gain (ADG) per piglets. The diarrhea occurrence in the piglets were observed daily and the diarrhea rate was calculated. The calculation formulae are listed as follows: ADFI (kg/d) = total feed intake/experimental period; ADG (kg/d) = (final weight - initial weight)/experimental period; diarrhea rate (%) = (number of piglets with diarrhea × number of days of diarrhea)/(total number of experiment piglets × experimental period) × 100%.

At experiment termination, all piglets were anesthetized by electronarcosis and then sacrificed. Blood samples were collected in heparin sodium anticoagulant tubes and centrifuged at 1500 × *g* for 10 min to obtain the plasma. The jejunum, ileum, tissues were collected stored at -80 °C for analysis. Partial jejunum and ileum tissues were treated with 4% paraformaldehyde or 2.5% glutaraldehyde for subsequent analysis. Cecal contents of piglets were collected and stored at -80 °C for 16S rRNA and short-chain fatty acids (SCFAs) analyses.

### Detection of serum D-Lactate and diamine oxidase (DAO)

The levels of D-Lactate and DAO in serum samples were determined using ELISA kits purchased from the Jiangsu Meimian Industrial Co., Ltd (Jiangsu, China). All experimental steps were carried out according to the kit instructions.

### Histopathology and ultrastructure analysis

After fixation in a 4% paraformaldehyde for 24 h, intestinal tissues were embedded in paraffin and sectioned at 5 μm with a microtome (Leica RM2016, Nussloch, Germany). Then, some of the slices were stained with hematoxylin and eosin (H&E) for jejunum and ileum morphological examination. The other slices were stained with periodic acid-Schiff for the determination of goblet cells in the ileum. The slices were visualized and photographed with microscope (Nikon, Tokyo, Japan).

After fixation in a 2.5% glutaraldehyde for 24 h, intestinal tissues were fixed with osmium tetroxide. Then, the tissues were dehydrated, embedded and sectioned. After staining with uranyl acetate and lead citrate. The tissues were visualized with transmission electron microscopy (TEM) (Hitachi H-7650, Tokyo, Japan).

### Immunohistochemistry analysis

After fixation in a 4% paraformaldehyde for 24 h, ileum tissues were embedded in paraffin and sectioned at 5 μm. And then, the slices were incubated with antibodies against MUC2, followed by incubation with the second antibody (Servicebio, Wuhan, China) and 4’, 6-Diamidino-2-phenylindole (DAPI). The slices were viewed under a fluorescence scanning microscope (Danjier, Shandong, China).

### Quantitative RT-PCR analysis of gene expression

Total RNA from jejunum and ileum was obtained and reverse-transcribed into cDNA. These steps were carried out using TRIzol Reagent and PrimeScript ™ RT reagent Kit with gDNA Eraser Kit (Takara, Beijing, China) according to the manufacturer’s instructions. The mRNA expression levels of genes related to tight junctions, mucins and TLR4/NF-κB signaling pathway in the jejunum and ileum were evaluated by a standard real-time polymerase chain reaction (RT-PCR) method as previously described [20, 24]. The mRNA expression was conducted with a commercially available kit (Takara, Beijing, China). The β-actin was served as a reference gene. Next, the cycle threshold (CT) values were applied to evaluate the relative quantification of gene expression. The primer sequences are shown in Table 2.

**Table 2:** Primer sequences used for RT-qPCR analysis (F: forward; R: reverse)

GenBank accession No.	Primer sequence (5' to 3')	Product length, bp
*TLR4* (NM_001293316.1)	F: CCTTTTCATCTCTGCCTTCACTAC	112
	R: GGGACACCACGACAATAACCT	
*MyD88* (NM_001099923.1)	F: CTCTGGCAGCGCTCAATGTG	105
	R: AGTTCATCTCCTCCGCCAGC	
*NF-κB* (NM_001114281.1)	F: CTGAGGCTATAACTCGCTTGGTGAC	131
	R: CATGTCCGCAATGGAGGAGAAGTC	
*TNF-α* (NM_214022.1)	F: GCACTGAGAGCATGATCCGAGAC	120
	R: CGACCAGGAGGAAGGAGAAGAGG	
*IL-1β* (NM_214055.1)	F: GCCAACGTGCAGTCTATGGAGTG	91
	R: GGTGGAGAGCCTTCAGCATGTG	
*IL-6* (NM_214399.1)	F: ATAAGGGAAATGTCGAGGCTGTGC R: GGGTGGTGGCTTTGTCTGGATTC	93
*IL-8* (NM_213867.1)	F: TCCAAACTGGCTGTTGCCTTCTTG R: GGGGTGGAAAGGTGTGGAATGC	132
*ZO-1* (XM_021098856.1)	F: TGGCATTATTCGCCTTCATAC R: AGCCTCATTCGCATTGTTT	171
Occludin (NM_001163647.2)	F: TCAGGTGCACCCTCCAGATT R: AGGAGGTGGACTTTCAAGAGG	118
Claudin-4 (NM_001161637.1)	F: CAACTGCGTGGATGATGAGA R: CCAGGGGATTGTAGAAGTCG	140
Claudin-1 (NM_001244539.1)	F: ATTTCAGGTCTGGCTATCTTAGTTGC R: AGGGCCTTGGTGTTGGGTAA	214
*JAM-A* (NM_001128444.1)	F: AATCAGTGTTCCCTCCTCTGCTAC R: ACGGTTGCTCTTGGGCTCT	136
*MUC1* (XM_001926883.5)	F: GTGCCGCTGCCCACAACCTG R: AGCCGGGTACCCCAGACCCA	141
*MUC2* (XM_013989745.1)	F: GGTCATGCTGGAGCTGGACAGT R: TGCCTCCTCGGGGTCGTCAC	181
β-actin (AY550069)	F: ATGCTTCTAGGCGGACTGT	211
	R: CCATCCAACCGACTGCT	

### Western blot analysis

The samples (100 mg) were harvested and lysed at 4 °C with 1 mL RIPA lysis buffer containing 1% phenylmethanesulfonyl fluoride (PMSF) (Beyotime Biotechnology, Shanghai, China), followed by protein concentration determination using a BCA protein assay kit (Beyotime Biotechnology, Shanghai, China). The protein was separated by 10% SDS-PAGE gradient gel, transferred to PVDF membranes (Millipore, Billerica, MA, USA) and blocked with 5% skim milk powder. The membranes were incubated with primary antibodies and then incubated with HRP-conjugated secondary antibodies. After washing with TBST 3 times, the membranes were detected with the BeyoECL Star kit (Beyotime Biotechnology, Shanghai, China) and placed in the gel imaging system (Uvitec, Cambridge, Britain). Images were analyzed by measuring the intensity of correctly sized bands using Alpha Imager 2200 (Alpha Innotech Corporation, CA, USA), and the protein expression levels were normalized to β-actin.

### 16S rDNA gene sequencing and bioinformatics analysis

Total microbial DNA from samples was obtained using HiPure Soil DNA Kit (Magen, Guangzhou, China) according to manufacturer’s instructions. DNA concentration was monitored by Qubit3.0 Fluorometer. After that, the specific primers with barcode were used to amplify the V3-V4 regions of 16S rDNA. The forward primers sequence is 5'-GCTACGGGNGGCWGCAG3', and the reverse primers is 5'-GGACTACHVGGGTATCTAAT-3'. Amplicons were purified and quantified using commercially available kit (Axygen Biosciences, CA, USA) and RT-PCR System (Life Technologies, Foster City, USA), respectively. After purification, the amplicons were collected in equimolar and added to sequencing adapters to generate sequencing libraries. After that, the paired-end sequencing was carried out on an Illumina platform, then the raw reads were obtained. For details, please refer to the previous study [25].

Raw reads were filtered and merged as raw tags using FASTP and FLASH, respectively. Then, the raw tags were further filtered to make the clean tags. After quality filter, the clean tags were used for clustering to obtained operational taxonomic units (OTUs). All chimeric tags were removed and finally obtained effective tags. After obtaining OTU, OTU abundance was conducted based on effective tags. Next, representative sequence in each OTU was found for species annotation and classification, and draw bar charts were drawn.

### Quantification of short-chain fatty acids (SCFAs) in cecal contents

20 mg cecal contents were weighed and placed in EP tube containing 1 mL phosphoric acid (0.5% v/v) solution. The samples were mixed and extracted for 10 min, and sonicated for 5 min, and centrifuged for 10 min at 6000 r/min and 4 °C. And 0.1 mL supernatant was taken and transferred to a centrifugal tube, and 0.5 mL MTBE (containing internal standard) solution was added. Then, the samples were vortexed for 3 min and sonicated for 5 min. After that, centrifuge for 10 min at 12,000 r/min and 4 °C. After centrifugation, 0.2 mL of supernatant was filter-sterilized with a 0.22-μm filter and absorbed into the sampling bottle for GC-MS/MS analysis.

### Statistical analysis

The data obtained from all experiment were performed with univariate (ANOVA) analyses with Tukey’s post-tests using SPSS 23.0 (SPSS, IL, USA) and GraphPad Prism 7 (GraphPad Software, CA, USA). The Kruskal-Wallis tests was used when the data did not conform to the normal distribution. The relative abundance of cecal microbiota at the genus level was compared between two groups and multiple groups using Wilcoxon rank sum test and Kruskal-Wallis test, respectively. Data are mean ± SEM. “*” means *P* < 0.05, “**” means *P* < 0.01, “***” means *P* < 0.001.

## Results

### Effect of LGG on growth performance of weaned piglets exposed to DON

**Fig. 1:**
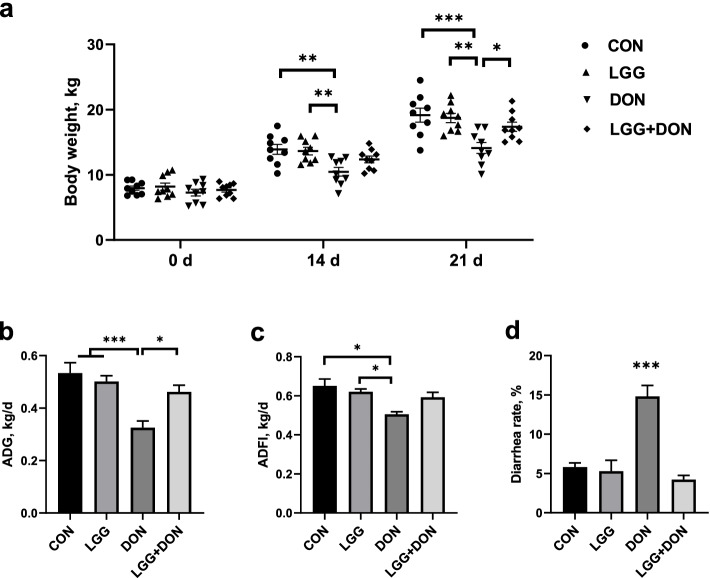
Effect of LGG on growth performance of weaned piglets exposed to DON. **a** Body weight, **b** Average daily gain (ADG), **c** Average daily feed intake (ADFI), and **d** Diarrhea rate from different groups were calculated (*n* = 9). CON, basal diet; LGG, basal diet supplemented with 1.77 × 10^11^ CFU/kg LGG; DON, DON-contaminated diet containing 3.11 mg/kg DON; and LGG + DON, DON-contaminated diet containing 3.11 mg/kg DON and 1.77 × 10^11^ CFU/kg LGG. Data are mean ± SEM. “*” means *P* < 0.05, “**” means *P* < 0.01, “***” means *P* < 0.001

Body weight, ADG, ADFI and diarrhea rate of piglets in the four groups were first monitored throughout the entire trial. As shown in Fig. 1a, the body weight of DON group at 14 d and 21 d were significantly reduced compared with the CON group. DON exposure induced decreases in ADG and ADFI compared to the CON group (Fig. 1b, c). By contrast, no significant increase of body weight, ADG and ADFI was observed of piglets in LGG + DON group as compared to CON group. DON exposure significantly increased the diarrhea rate of piglets compared to the other three groups (Fig. 1d). Piglets in the LGG + DON group had lower diarrhea rate than those in the DON group. Taken together, supplementation of LGG into the DON-contaminated diet could effectively relieve the growth inhibition of piglets induced by DON.

### Effect of LGG on jejunum and ileum morphology of weaned piglets exposed to DON

**Fig. 2:**
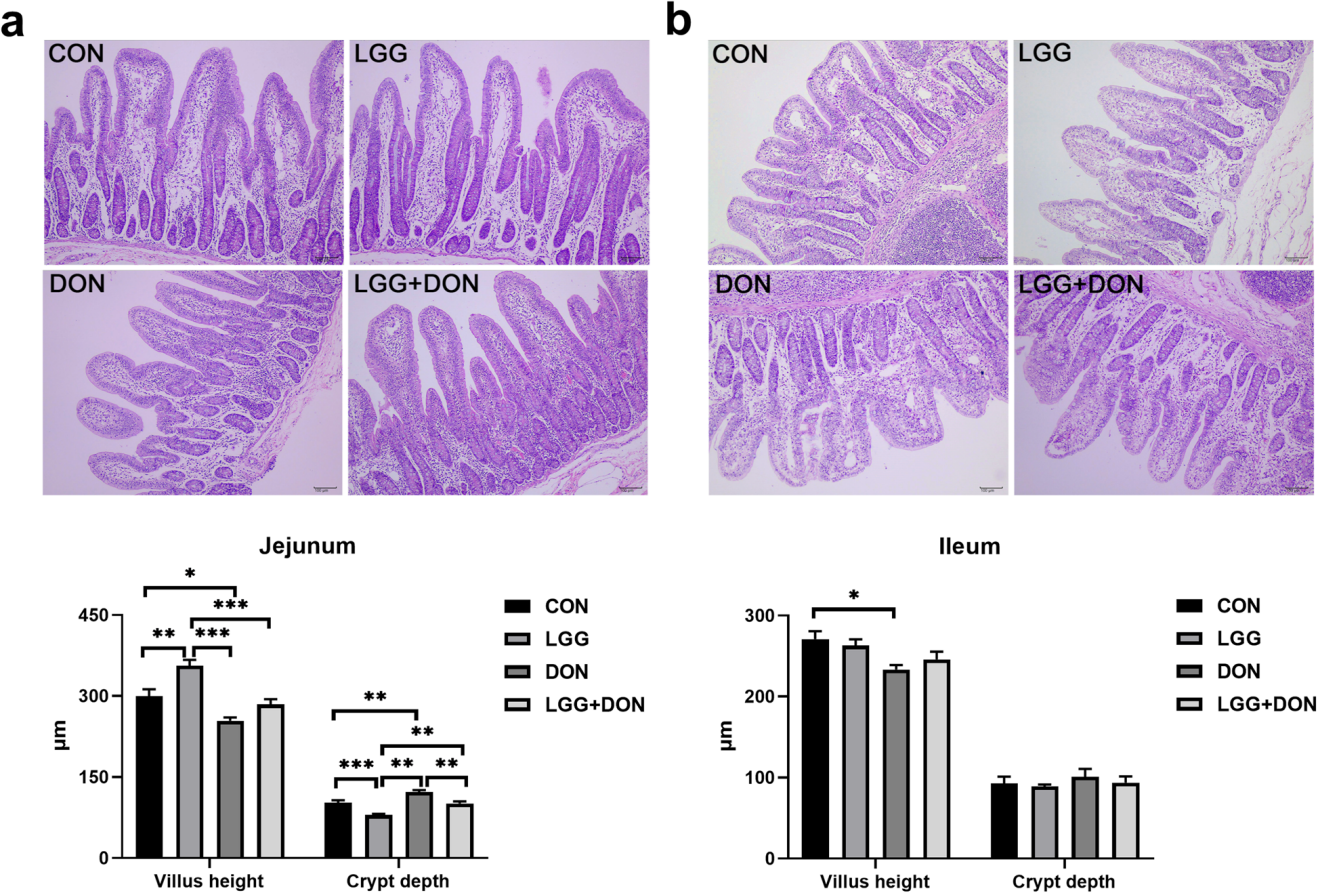
Effect of LGG on jejunum and ileum morphology of weaned piglets exposed to DON. **a** Representative images of hematoxylin and eosin-stained of jejunum and ileum sections (*n* = 6). Scale bar = 200 μm. **b** Villus height and crypt depth in the jejunum and ileum of piglets from different groups were quantified (*n* = 6). CON, basal diet; LGG, basal diet supplemented with 1.77 × 10^11^ CFU/kg LGG; DON, DON-contaminated diet containing 3.11 mg/kg DON; and LGG + DON, DON-contaminated diet containing 3.11 mg/kg DON and 1.77 × 10^11^ CFU/kg LGG. Data are mean ± SEM. “*” means *P* < 0.05, “**” means *P* < 0.01, “***” means *P* < 0.001

Histological analysis indicated that DON exposure significantly reduced villus height and increased crypt depth in jejunum compared with CON group (Fig. 2). A significant increase in jejunal villus height and a decrease in jejunal crypt depth were observed in the LGG group compared with CON group. Compared to the DON group, the crypt depth in jejunum were decreased in the LGG + DON group. In addition, piglets exposed to DON significantly reduced villus height in ileum. There were no significant alterations in ileum villus height among the CON, LGG, and LGG + DON groups. No significant differences in the ileum crypt depth of piglets were observed between the four treatment groups. Overall, these results suggest that supplementation of LGG into the DON-contaminated diet attenuates DON-induced intestinal damage by increasing villus height in jejunum and ileum, reducing crypt depth in jejunum.

### Effect of LGG on the ultrastructure of jejunum in weaned piglets exposed to DON

**Fig. 3:**
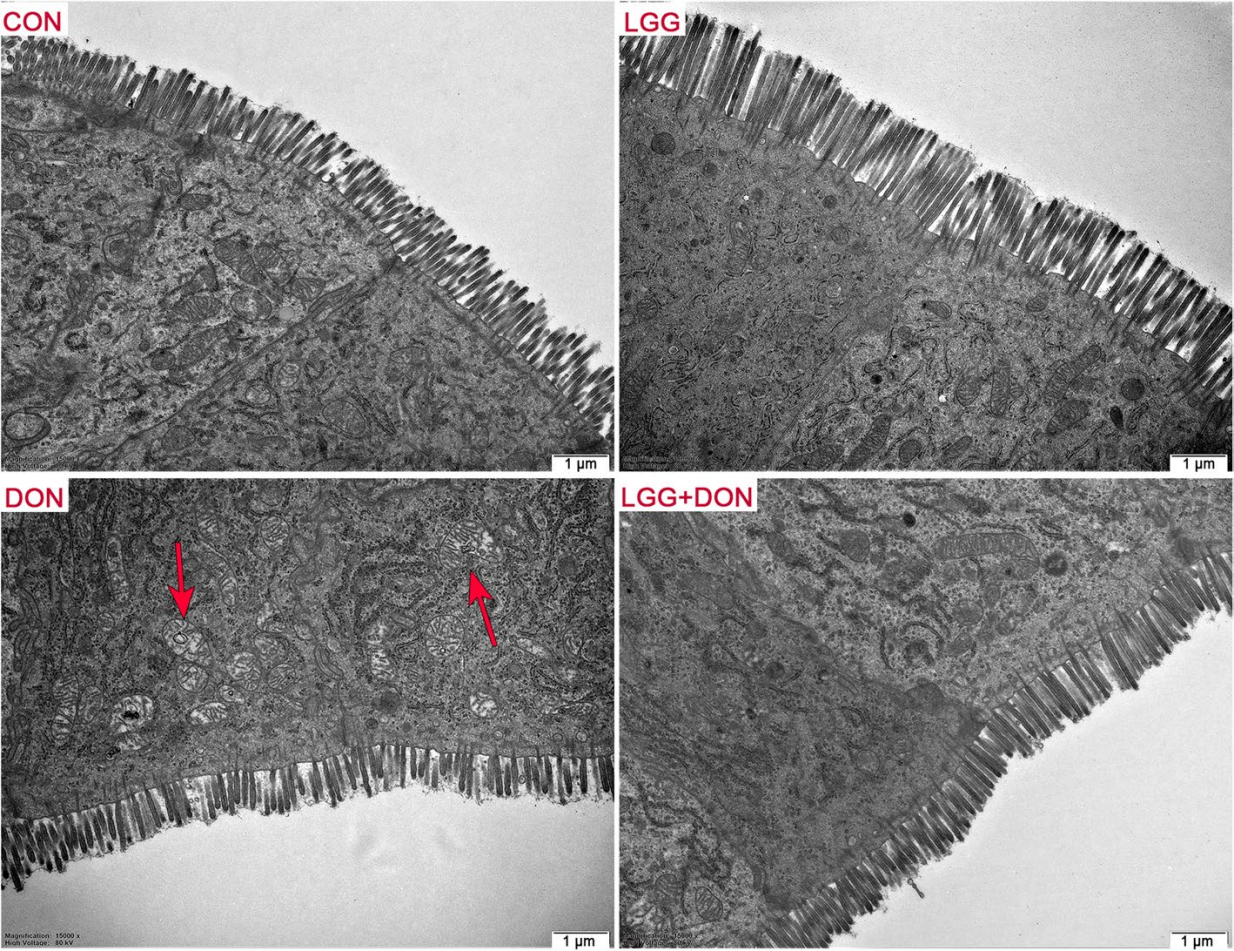
Effect of LGG on the ultrastructure of jejunum in weaned piglets exposed to DON. Representative TEM pictures of jejunum in four groups (magnification ×15,000). Red arrows, mitochondria swelling. CON, basal diet; LGG, basal diet supplemented with 1.77 × 10^11^ CFU/kg LGG; DON, DON-contaminated diet containing 3.11 mg/kg DON; and LGG + DON, DON-contaminated diet containing 3.11 mg/kg DON and 1.77 × 10^11^ CFU/kg LGG. Data are mean ± SEM. “*” means *P* < 0.05, “**” means *P* < 0.01, “***” means *P* < 0.001

TEM observation of the ultrastructure of jejunum in weaned piglets was shown in Fig. 3. CON and LGG groups showed regular jejunum morphology, clear cell boundaries, neatly arranged microvilli and clear cristae mitochondria. In contrast, shorten microvilli, swollen mitochondria with broken and vague cristae were observed in the DON group. However, these alterations were not seen in LGG + DON group. Collectively, the DON-induced ultrastructure alteration of jejunum in piglets was alleviated by the LGG supplementation.

### Effect of LGG on activities of plasma DAO and D-lactate of weaned piglets exposed to DON

**Fig. 4:**
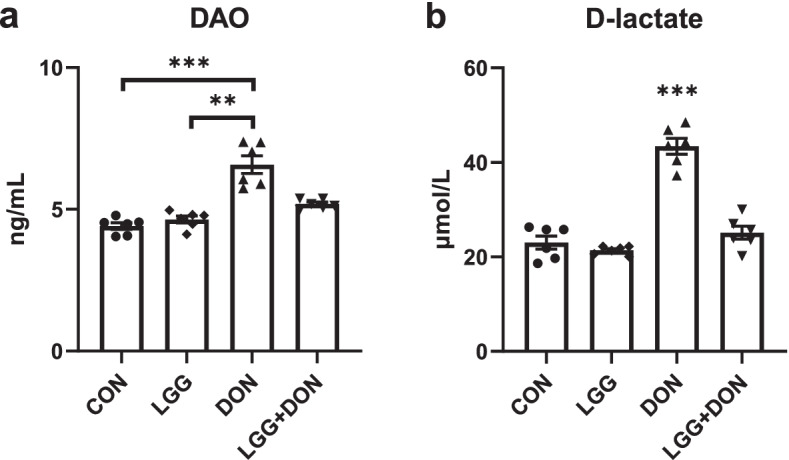
Effect of LGG on activities of plasma DAO and D-lactate of weaned piglets exposed to DON. **a** DAO activity, and **b** D-lactate level in the plasma of piglets (*n* = 6). CON, basal diet; LGG, basal diet supplemented with 1.77 × 10^11^ CFU/kg LGG; DON, DON-contaminated diet containing 3.11 mg/kg DON; and LGG + DON, DON-contaminated diet containing 3.11 mg/kg DON and 1.77 × 10^11^ CFU/kg LGG. Data are mean ± SEM. “*” means *P* < 0.05, “**” means *P* < 0.01, “***” means *P* < 0.001

DON exposure had higher plasma concentration of DAO than that in the CON group (Fig. 4a). No significant differences in the activity of plasma DAO were noted between the LGG + DON group and the DON group, but the LGG + DON group showed a decrease compared with the DON group and had no significant difference with CON group. Compared with CON group, DON exposure induced significant increases in plasma D-lactate concentration (Fig. 4b). There were no significant alterations in plasma D-lactate concentration among the CON, LGG, and LGG + DON groups.

### Effect of LGG on goblet cells and MUC2 of weaned piglets exposed to DON

**Fig. 5:**
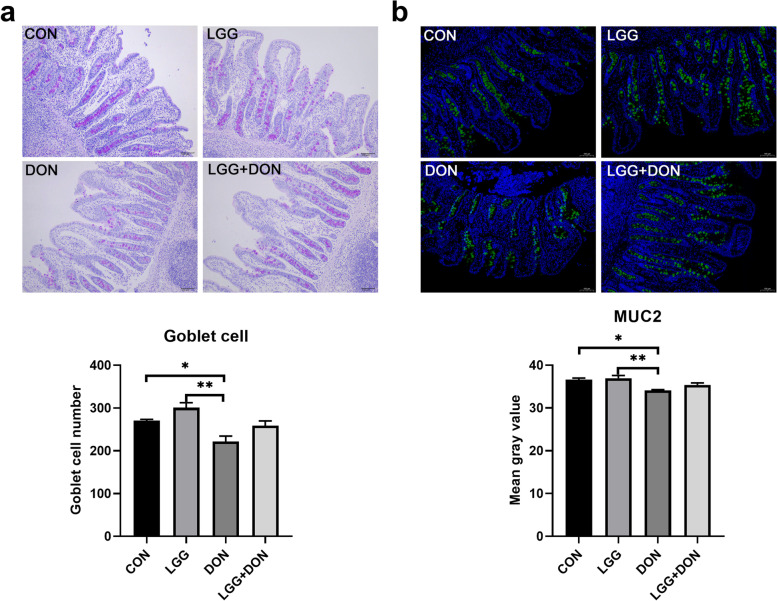
Effect of LGG on goblet cells and MUC2 of weaned piglets exposed to DON. **a** Representative images of periodic acid-schiff (PAS) staining (magnification × 100, scale bar 100 μm) of ileum sections and the number of goblet cells in ileum (*n* = 6). **b** Representative images of immunohistochemical staining of MUC2 (green) and nuclei (blue) (magnification ×100, scale bar 100 μm) and mean gray value of MUC2 in different groups (*n* = 6). CON, basal diet; LGG, basal diet supplemented with 1.77 × 10^11^ CFU/kg LGG; DON, DON-contaminated diet containing 3.11 mg/kg DON; and LGG + DON, DON-contaminated diet containing 3.11 mg/kg DON and 1.77 × 10^11^ CFU/kg LGG. Data are mean ± SEM. “*” means *P* < 0.05, “**” means *P* < 0.01, “***” means *P* < 0.001

To evaluate the effect of LGG on the intestinal barrier function of weaned piglets exposed to DON, the goblet cells number and the expression of MUC2 in ileum of piglets were performed (Fig. 5). DON exposure reduced the number of goblet cells and the expression of MUC2 in ileum of piglets. There was an increase in the number of goblet cells in LGG group compared with CON group but not significantly. Notably, no significant differences in the number of goblet cells and the expression of MUC2 were noted between the CON group and the LGG + DON group, which indicated that LGG played a role in the maintaining intestinal barrier function of weaned piglets exposed to DON.

### Effect of LGG on intestinal barrier function and TLR4/NF-κB signaling pathway of weaned piglets exposed to DON

**Fig. 6:**
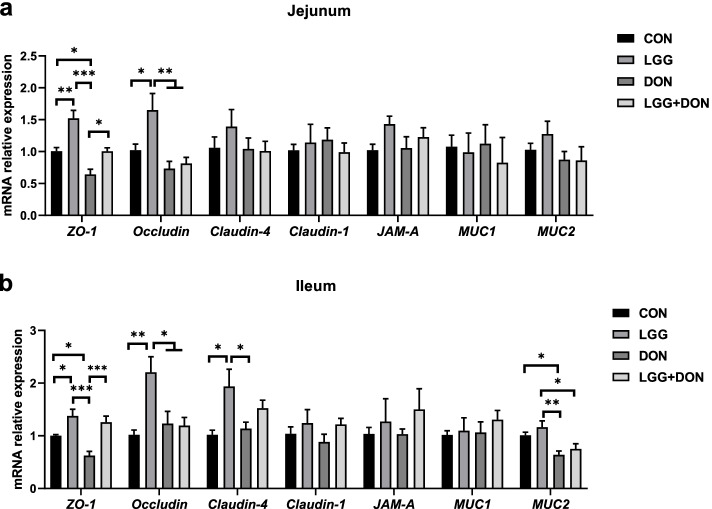
Effect of LGG on the expression of genes related to tight junctions and mucins in the jejunum and ileum of weaned piglets exposed to DON. **a**, **b** Relative mRNA expression levels of *ZO-1*, occludin, Claudin-4, Claudin-1, *JAM-A*, *MUC1* and *MUC2* in the jejunum and ileum assessed by real-time PCR (*n* = 8). β-actin was used as an internal control. CON, basal diet; LGG, basal diet supplemented with 1.77 × 10^11^ CFU/kg LGG; DON, DON-contaminated diet containing 3.11 mg/kg DON; and LGG + DON, DON-contaminated diet containing 3.11 mg/kg DON and 1.77 × 10^11^ CFU/kg LGG. Data are mean ± SEM. “*” means *P* < 0.05, “**” means *P* < 0.01, “***” means *P* < 0.001

To further investigate the role of LGG in intestinal damage induced by DON, the expression of genes related to tight junctions and mucins in the jejunum and ileum of piglets were analyzed (Fig. 6). DON exposure significantly decreased the mRNA expression of *ZO-1* in jejunum and ileum compared to the CON group, while supplementation of LGG into the DON-contaminated diet increased the levels of *ZO-1* in jejunum and ileum compared to the DON group. The levels of *ZO-1* and Occludin in jejunum and ileum and the levels of Claudin-4 in ileum were higher in the LGG group than the CON group. A significant decrease in the ileum mRNA expression of *MUC2* was observed in DON group compared to the CON group. The jejunal mRNA level of *MUC2* was higher in the LGG + DON group than the DON group, but there were no significant differences.

**Fig. 7:**
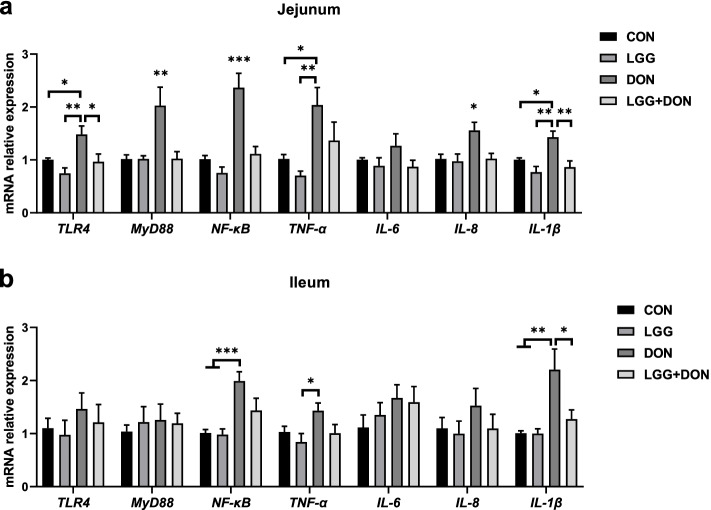
Effect of LGG on the expression of related genes in TLR4/NF-κB signaling pathway in the jejunum and ileum of weaned piglets exposed to DON. **a**, **b** Relative mRNA expression levels of *TLR4*, *MyD88*, *NF-κB*, *TNF-α*, *IL-6*, *IL-8* and *IL-1β* in the jejunum and ileum assessed by real-time PCR (*n* = 8). β-actin was used as an internal control. CON, basal diet; LGG, basal diet supplemented with 1.77 × 10^11^ CFU/kg LGG; DON, DON-contaminated diet containing 3.11 mg/kg DON; and LGG + DON, DON-contaminated diet containing 3.11 mg/kg DON and 1.77 × 10^11^ CFU/kg LGG. Data are mean ± SEM. “*” means *P* < 0.05, “**” means *P* < 0.01, “***” means *P* < 0.001

**Fig. 8:**
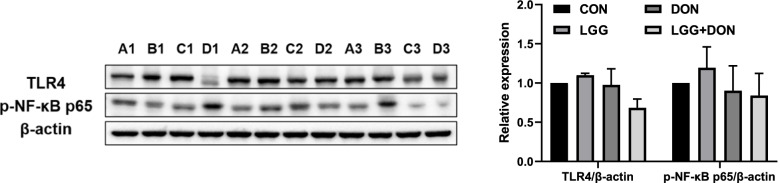
Effect of LGG on the relative protein expression of TLR4 and p-NF-κB p65 in the jejunum of weaned piglets exposed to DON. β-actin was used as an internal control. CON, basal diet (A1, A2, A3); LGG, basal diet supplemented with 1.77 × 10^11^ CFU/kg LGG (B1, B2, B3); DON, DON-contaminated diet containing 3.11 mg/kg DON (C1, C2, C3); and LGG + DON, DON-contaminated diet containing 3.11 mg/kg DON and 1.77 × 10^11^ CFU/kg LGG (D1, D2, D3). Data are mean ± SEM

In addition, the TLR4/NF-κB signaling pathway were examined in jejunum and ileum (Fig. 7). DON exposure enhanced the expression of *TLR4*, *MyD88* and *NF-κB* in jejunum compared to the other three treatments. These results indicated that DON stimulated the TLR4/NF-κB signaling pathway, resulting in the production of numerous proinflammatory molecules (*TNF-α*, *IL-8* and *IL-1β*) in jejunum. Compared to CON group, piglets exposed to DON showed a significant increase in the mRNA expression of *NF-κB* and *IL-1β* in ileum. Conversely, compared to DON group, the mRNA expression of *TLR4*, *MyD88*, *NF-κB*, *IL-8* and *IL-1β* in the jejunum were significantly decreased in the LGG + DON group. A significant decrease in the ileum mRNA expression of *IL-1β* was observed in LGG + DON group compared to DON group. In addition, no statistically significant difference was detected in the relative protein expression of TLR4 and p-NF-κB p65 in the jejunum between treatments (Fig. 8). Collectively, these findings show that supplementation of LGG into the DON-contaminated diet ameliorates DON-induced intestinal toxicity in piglets by increasing the expression of tight junctions and mucins and regulating TLR4/NF-κB signaling pathway.

### Effect of LGG on cecal microbiota of weaned piglets exposed to DON

**Fig. 9:**
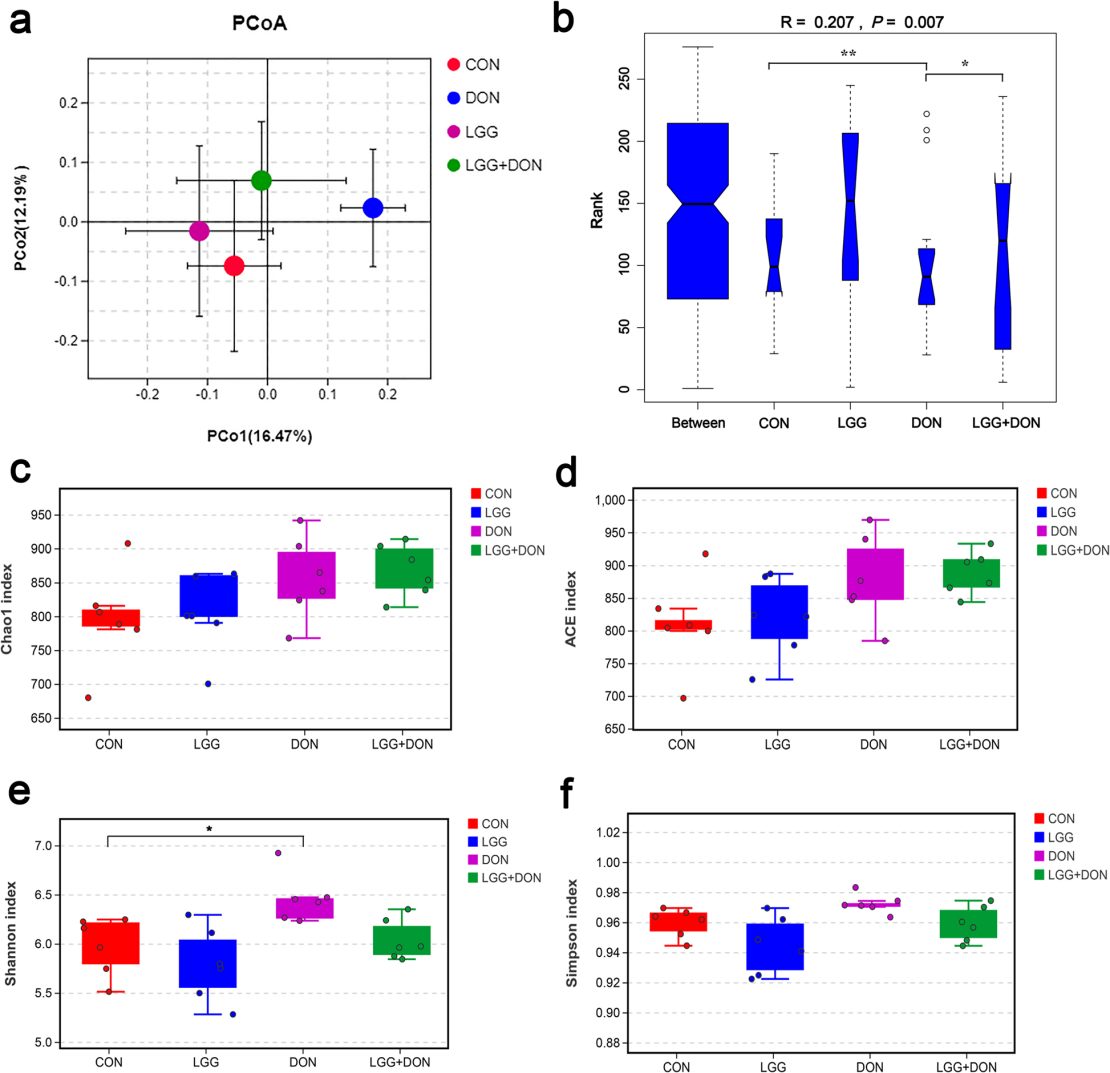
Effect of LGG on α and β diversity of cecal contents in weaned piglets exposed to DON. **a** Principal coordinate analysis (PCoA) plot of the cecal microbiota composition at the operational taxonomic unit (OTU) level from different groups. Points with different colors represent the centroid of each group. The closer the points, the more similar the gut microbiota structure. **b** The significant differences between groups were calculated by analysis of similarity (ANOSIM) analysis. The vertical axis of the box plot represents the distance ranking, the horizontal axis between represents the distance between groups, and the other represents the distance within the corresponding group. The R value indicates the degree of difference between and within groups. *P* value indicates the significance of differences between and within groups. **c-f** Diversity and richness indexes of cecal microbiota in each group. CON, basal diet; LGG, basal diet supplemented with 1.77 × 10^11^ CFU/kg LGG; DON, DON-contaminated diet containing 3.11 mg/kg DON; and LGG + DON, DON-contaminated diet containing 3.11 mg/kg DON and 1.77 × 10^11^ CFU/kg LGG. Data are mean ± SEM, *n* = 6. “*” means *P* < 0.05, “**” means *P* < 0.01, “***” means *P* < 0.001

**Fig. 10:**
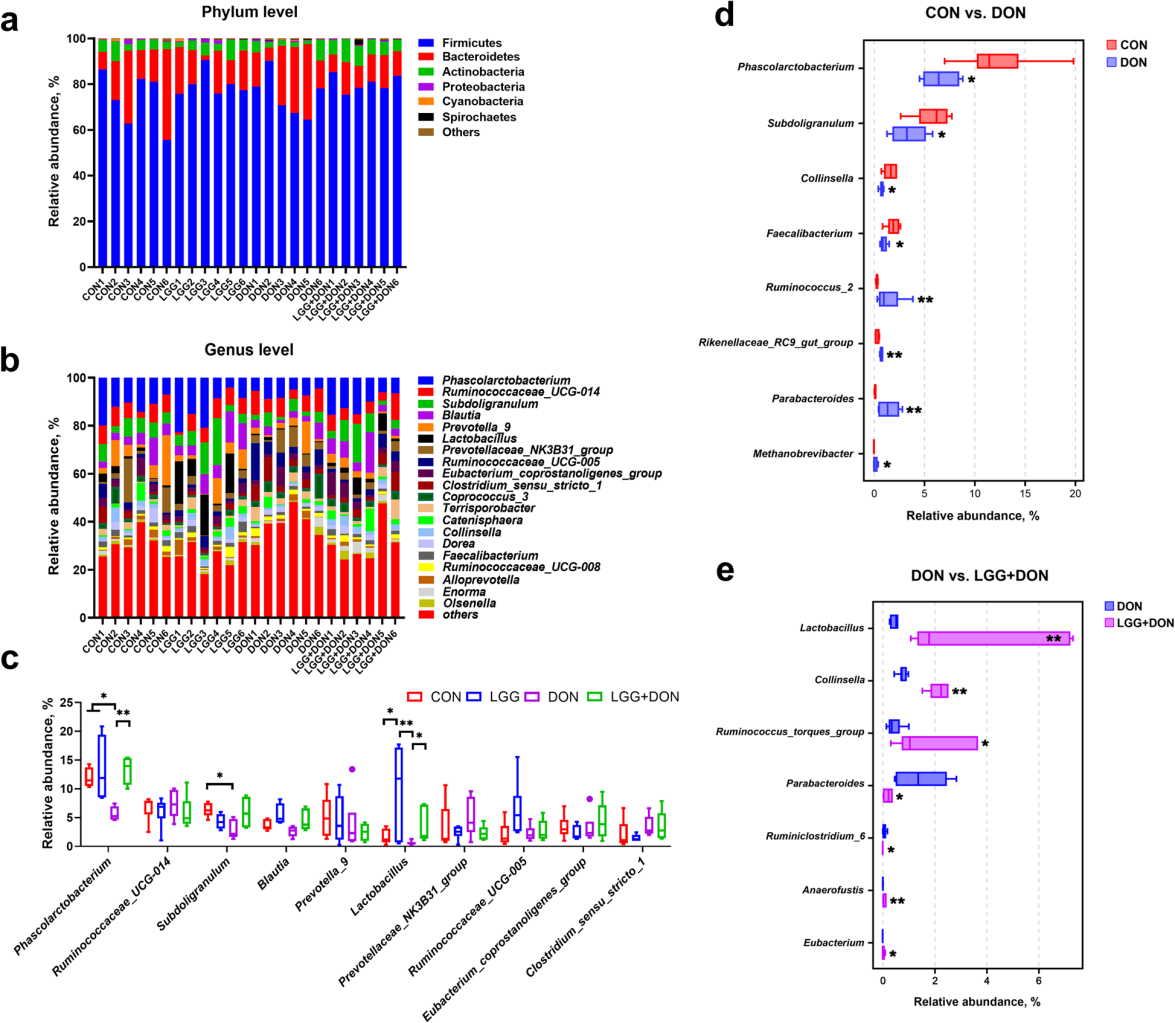
Effect of LGG on cecal microbiota composition of weaned piglets exposed to DON. **a** Relative abundance of cecal microbiota at the phylum levels. **b** Relative abundance of cecal microbiota at the genus levels. **c** Relative abundance of cecal microbiota at the genus level (top 10) in the different groups. **d**, **e** Relative abundance of cecal microbiota with significant differences of the comparison groups CON vs. DON and DON vs. LGG + DON at the genus levels. CON, basal diet; LGG, basal diet supplemented with 1.77 × 10^11^ CFU/kg LGG; DON, DON-contaminated diet containing 3.11 mg/kg DON; and LGG + DON, DON-contaminated diet containing 3.11 mg/kg DON and 1.77 × 10^11^ CFU/kg LGG. Data are mean ± SEM, *n* = 6. “*” means *P* < 0.05, “**” means *P* < 0.01, “***” means *P* < 0.001

To explore the effect of LGG on gut microbiota of weaned piglets exposed to DON, we analyzed the cecal microbiota composition by performing a 16S rDNA gene sequencing. Principal coordinate analysis (PCoA) analysis showed the DON group was a clear separated from the other groups (Fig. 9a). The ANOSIM analysis based on Bray-Curtis distances revealed a significant difference in microbial communities among the groups (Fig. 9b). The results of the α diversity analysis revealed DON increased the Shannon index of gut microbiota composition, but no effect could be found on the Chao1 index, ACE index and Simpson index compared with the CON group (Fig. 9c-f). LGG did not have a significant impact on diversity and richness indexes of cecal microbiota (Fig. 9c-f). The gut bacterial composition of piglets was dominated by Firmicutes, Bacteroidetes and Actinobacteria at the phylum level (Fig. 10a). The relative abundances of cecal microbiota at the genus level were different among groups (Fig. 10b-e). By using the Kruskal-Wallis test analysis, a significant decrease in the relative abundances of *Phascolarctobacterium* and *Subdoligranulum* were observed in DON group compared to the CON group, but this difference was not seen in the LGG + DON group. The relative abundances of *Lactobacillus* were higher in the LGG group than the CON group. Compared with the DON group, the relative abundance of *Lactobacillus* in the LGG + DON group was significantly increased. The Wilcoxon rank sum test analysis results showed that compared to CON group, DON exposure significantly decreased the relative abundances of *Phascolarctobacterium*, *Subdoligranulum*, *Collinsella* and *Faecalibacterium*, while increasing the relative abundances of *Ruminococcus_2*, *Rikenellaceae_RC9_gut_group*, *Parabacteroides* and *Methanobrevibacter*. In addition, the relative abundances of *Lactobacillus*, *Collinsella*, *Ruminococcus_torques_group*, *Anaerofustis* and *Eubacterium* were significantly increased in the LGG + DON group, whereas the relative abundances of *Parabacteroides* and *Ruminiclostridium_6* were significantly decreased compared to the DON group. These data suggest that DON exposure altered the cecal microbiota structure of piglets, and supplementation with LGG could improve gut health by increasing beneficial microbe.

### Effect of LGG on cecal SCFAs of weaned piglets exposed to DON

**Fig. 11:**
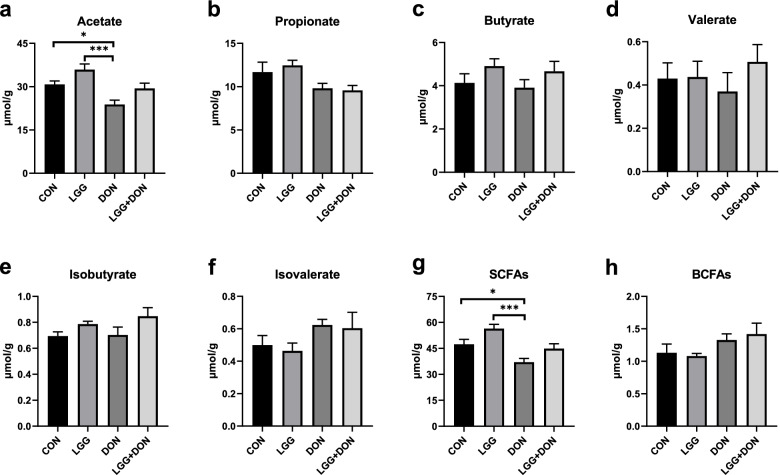
Effect of LGG on the cecal short-chain fatty acids (SCFAs) of weaned piglets exposed to DON. **a** Acetate, **b** Propionate, **c** Butyrate, **d** Valerate, **e** Isobutyrate, **f** Isovalerate, **g** SCFAs, and **h** BCFAs was quantified (*n* = 8). CON, basal diet; LGG, basal diet supplemented with 1.77 × 10^11^ CFU/kg LGG; DON, DON-contaminated diet containing 3.11 mg/kg DON; and LGG + DON, DON-contaminated diet containing 3.11 mg/kg DON and 1.77 × 10^11^ CFU/kg LGG. Data are mean ± SEM. “*” means *P* < 0.05, “**” means *P* < 0.01, “***” means *P* < 0.001

To explore the changes in metabolites caused by the gut microbiota remodeled by DON and LGG, the concentration of SCFAs in the cecal content of piglets were analyzed, including acetate, propionate, butyrate, valerate, isobutyrate, isovalerate, SCFAs and branch-chain fatty acids (BCFAs). The results showed that the concentration of acetate and SCFAs in DON group were significantly decreased compared to the CON group (Fig. 11a, g). However, there is no significant difference in the concentrations of acetate and SCFAs in the LGG + DON group as compared to CON group and DON group. In addition, no significant differences were observed in the concentrations of propionate, butyrate, valerate, isobutyrate, isovalerate, and BCFAs among the four groups (Fig. 11b-f, h). These data suggest that supplementation of LGG into the DON-contaminated diet can improve the reduction of acetate and SCFAs induced by DON.

## Discussion

DON contamination is a worldwide problem, which has attracted the attention of many researchers. No matter in developed countries or developing countries, the high incidence rate of DON in wheat and wheat flour samples was reported [26], posing a serious threat to the safety of humans and animals. DON has toxic effects on animals and human beings, causing anorexia, emesis, growth suppression, and intestinal inflammation [5, 27]. Hence, an urgent demand exists for researchers to find a new substance to counteract harmful effects of DON. LGG, one of the probiotics, is a promising candidate because of its beneficial effects on intestinal functions [28]. In this work, the effects of LGG on the intestinal injury induced by DON exposure were comprehensively evaluated from the perspectives of intestinal barrier function, intestinal inflammation, and intestinal microbes.

In the current study, DON significantly decreased the growth performance of piglets, which is in accordance with the findings of previous studies [9, 29]. Interestingly, supplementation of LGG is able to restore the adverse effect induced by DON exposure, as evidenced by increasing body weight at 14 d and 21 d, enhancing ADG and ADFI and decreasing diarrhea rate. Studies have shown that LGG is not only able to alleviate the intestinal inflammation of piglets induced by *Salmonella Infantis* [30], but is also able to improve intestinal barrier function of piglets challenged with Lipopolysaccharide [18]. Therefore, we speculate that LGG may enhance growth performance of piglets exposed to DON by improving intestinal health. In order to confirm this assumption, the intestinal morphology was analyzed. The intestinal morphology is an important indicator reflecting the healthy state of intestine. The villus height, crypt depth and microvilli height are closely related to the digestion and absorption efficiency and growth performance [31]. In the present study, DON significantly decreased the villus height and increased crypt depth but dietary supplementation with LGG significantly decreased the crypt depth of the jejunum compared with CON group. In addition, DON also affects the ultrastructure of jejunum, which is evidenced by shorten microvilli, swollen mitochondria with broken and vague cristae. These effects were not identified in LGG + DON group. Therefore, the decrease of growth performance caused by DON may be due to the decrease of villus height and the increase of crypt depth, while LGG may improve growth performance of piglets exposed to DON by increasing the villus height and reducing the crypt depth of jejunum and ultimately increasing the absorption and utilization of nutrients in the small intestine.

The DAO and D-lactate are the most common indicators to evaluate intestinal barrier function [32]. In the present paper, the plasma concentrations of DAO and D-lactate were significant increased after piglets ingested DON-contaminated diet, indicating impaired intestinal barrier function. Similar findings were observed by other researchers [33, 34]. Remarkably, supplementation of LGG into the DON-contaminated diet had lower plasma concentrations of D-lactate than that in the DON group and had no significant difference with CON group. These data indicate that LGG played a role in alleviating the injury of intestinal barrier function induced by DON.

Goblet cell is a secretory cell that can synthesize and secrete mucus to form a mucous barrier to protect epithelial cells [35]. Previous studies have shown that DON can reduce the number of intestinal goblet cells, destroy the mucosal barrier and cause intestinal injury in piglets [34], which is also found in the present study. Notably, the present study showed supplementation of LGG into the DON-contaminated diet had higher, albeit non-significant, goblet cells number and mucin MUC2 expression in the ileum compared to DON group. MUC2 mucin is produced and secreted by intestinal goblet cells and its expression is closely related to the number of goblet cells [36]. In addition, the decreased mRNA expression of *MUC2* in ileum was found in DON group and likely accounts for the decreased levels of MUC2 mucin.

Intestinal barrier have functions other than maintaining intestinal homeostasis and have hindering pathogenic bacteria and toxins. However, disruption of the intestinal barrier results in loss of transport function and intestinal inflammation [37]. In the present work, DON exposure destroyed the intestinal barrier, as revealed by decreased mRNA expression of *ZO-1* in jejunum and ileum. ZO-1 is one of the intestinal barrier proteins, which also includes the occludin, claudins and JAM-A [37, 38]. Interestingly, the present study showed that LGG can improve intestinal barrier function, which was consistent with prior studies [39]. In addition, compared to DON group, supplementation of LGG into the DON-contaminated diet increased the mRNA expression of *ZO-1* in jejunum and ileum. These data indicate that supplementation of LGG into the DON-contaminated diet improves gut barrier function impaired by DON.

To further investigate the role of LGG in intestinal injury induced by DON, the expression of related genes in TLR4/NF-κB signaling pathway were examined in jejunum and ileum. Studies have shown that the TLR4/NF-κB signaling pathway is involved in a variety of inflammatory responses [40–42]. A previous study showed that DON exposure significantly activated the TLR4/NF-κB pathway [11], which can induce induces the production of numerous proinflammatory molecules (e.g., *TNF-α*, *IL-8* and *IL-1β*) [43]. This is in agreement with experimental data of piglets in this study. However, the present finding that the protein expression of TLR4 and p-NF-κB p65 in the jejunum was not different among the four groups, which was inconsistent with the mRNA expression results. This is possibly due to mRNA expression could not represent protein expression totally [44] and post-transcriptional regulation contributes substantially more to protein level changes than immediate changes induced by mRNA [45]. In addition, studies have shown *TLR4* deficiency attenuated tissue injury and decreased inflammatory response [41, 46]. One report has indicated that a novel soluble protein derived from LGG can inhibit the production of inflammation by suppressing the *TLR4*/*MyD88*/*NF-κB* axis [47]. Here we observed a similar finding that LGG ameliorates DON-induced intestinal inflammation through inhibiting the TLR4/NF-κB signaling pathway.

The gut microbiota plays an important role in maintaining intestinal barrier function and immune balance [48, 49]. Microbiota dysbiosis is closely related to intestinal inflammation and a kind of diseases [50]. Previous investigations showed that DON has a great influence on the composition and structure of the gut microbiota [7, 34]. In the present study, PCoA and ANOSIM analysis revealed that there were significant changes in the composition of the gut microbiota after DON exposure. Additionally, we observed an increased Shannon index in DON group compared with the CON group. In α-diversity analysis, Chao1 and ACE index represent the community richness, and Shannon and Simpson index represent community diversity. A previous study has also shown that DON increased the Shannon index [51], which is similar to this study. However, there are some inconsistent reports. One study reported that DON had no effect on the α-diversity of gut microbiota composition [7], and another reported that DON reduced the α-diversity of gut microbiota composition [52]. This inconsistency may be because DON disturbs the balance of gut microbiota, resulting in an increase in harmful bacteria and an increase in community diversity. Experimental conditions and other unidentified factors may also have contributed.

By using the Kruskal-Wallis tests and Wilcoxon rank sum test analysis, a significant increase in the relative abundances of *Ruminococcus_2*, *Rikenellaceae_RC9_gut_group*, *Parabacteroides* and *Methanobrevibacter* were observed in DON group compared to the CON group. Previous studies have shown that *Ruminococcus_2* and *Parabacteroides* are correlated with inflammation [53, 54], and *Rikenellaceae_RC9_gut_group*, *Methanobrevibacter* are associated with various diseases [55, 56]. This means that DON exposure increases the relative abundance of harmful bacteria, which may be one of the reasons for intestinal inflammation. Furthermore, a significant decrease in the relative abundances of *Collinsella*, *Faecalibacterium*, *Phascolarctobacterium*, *Subdoligranulum* were observed in DON group compared to the CON group. *Collinsella* is one of the beneficial genera and has been found in lower concentrations in irritable bowel syndrome patients [57–59]. Remarkably, the relative abundance of *Collinsella* was significantly increased after supplementation of LGG into the DON-contaminated diet compared to the DON group. These findings show that LGG may have a beneficial effect on DON-induced microbiota dysbiosis. Other studies have shown similar findings. LGG alleviates allergic airway inflammation [60] and improve survival from lipopolysaccharides-induced sepsis [61] by regulating the imbalance in the gut microbiota. In addition, *Phascolarctobacterium*, *Subdoligranulum*, *Faecalibacterium* and *Eubacterium* are SCFAs producers [62–65]. SCFAs produced by gut microbiota metabolism are the main energy source of intestinal cells. Furthermore, increasing evidence shows that SCFAs can regulate intestinal inflammation and improve intestinal barrier function [56, 66]. In fact, a decreased level of SCFAs was observed in DON group, while non-significant level of SCFAs were observed in LGG + DON group compared to the DON group. This is probably because DON reduced the abundance of SCFA-producing bacteria (e.g., *Phascolarctobacterium*, *Subdoligranulum* and *Faecalibacterium*), while LGG supplementation increased the relative abundance of SCFA producing bacteria (e.g., *Eubacterium*). In addition, cytokines are well known to be involved in the immune response. For the process of the immune response, the inflammation response usually occurs first [67]. In the present work, the ADG, ADFI and the mRNA expression of *IL-1β* in LGG + DON group were higher than those in the DON group. Interestingly, *Ruminococcus_torques_group* may be related to improving the growth performance and immunity [68]. A significant increase in the relative abundances of *Ruminococcus_torques_group* were observed in LGG + DON group compared to the DON group. This suggests that supplementation of LGG into the DON-contaminated diet alleviated the effects of DON growth performance and suppressed intestinal inflammation by increasing the relative abundances of *Ruminococcus_torques_group*. *Anaerofustis* and *Lactobacillus* are well known as beneficial bacterium [69, 70]. Specially, *Ruminiclostridium_6* is significantly positively correlated with pro-inflammatory cytokines, such as *IL-6* and *TNF-α* [71]. Here in this article supplementation of LGG into the DON-contaminated diet increased the relative abundances of beneficial bacteria such as *Collinsella*, *Lactobacillus*, *Ruminococcus_torques_group* and *Anaerofustis*, and decreased the relative abundances of harmful bacteria such as *Parabacteroides* and *Ruminiclostridium_6*. Meanwhile, gut microbiota as a biological barrier is closely linked with gut barrier integrity and intestinal inflammation. Previous studies showed LGG increased the intestinal permeability and promoted the immunologic barrier through regulating antimicrobial peptides and cytokines via TLR [17], which is similar with the present study. Therefore, LGG may improve intestinal barrier function by remodeling the intestinal microbial structure. Collectively, these data suggest that supplementation of LGG into the DON-contaminated diet can improve microbiota dysbiosis induced by DON, and promote the production of SCFAs, thereby contribute to improve intestinal health.

## Conclusions

The present study demonstrated that dietary LGG supplementation can protect against DON-induced intestinal injury in piglets. On one hand, the protective effect is may be obtained by improving the intestinal barrier function, and alleviating the intestinal inflammation through inhibiting the TLR4/NF-κB signaling pathway, which will further improve growth performance of piglets. On the other hand, the protective effect may be achieved through increasing the relative abundances of beneficial bacteria (e.g., *Collinsella*, *Lactobacillus*, *Ruminococcus_torques_group* and *Anaerofustis*), and decreasing the relative abundances of harmful bacteria (e.g., *Parabacteroides* and *Ruminiclostridium_6*), and promoting the production of SCFAs, which leads to improved intestinal health. The data of the experiments highlight the potential use of LGG as a probiotic to alleviate the adverse effects induced by DON exposure.

## Data Availability

The datasets produced and/or analyzed during the current study are available from the corresponding author on reasonable request.

## Abbreviations

DON: Deoxynivalenol
LGG: *Lactobacillus rhamnosus* GG
ADFI: Average daily food intake
ADG: Average daily gain
H&E: Hematoxylin–eosin
SCFAs: Short-chain fatty acids
TEM: Transmission electron microscopy
DAO: Diamine oxidase
IL-1β: Interleukin-1β
IL-6: Interleukin-6
IL-8: Interleukin-8
TNF-α: Tumor necrosis factor α
TLR4: Toll-like receptor 4
MyD88: Myeloid differentiation primary response 88
NF-κB: Nuclear factor-κB
